# The search for a melanoma-tailored chemotherapy in the new era of personalized therapy: a phase II study of chemo-modulating temozolomide followed by fotemustine and a cooperative study of GOIM (Gruppo Oncologico Italia Meridionale)

**DOI:** 10.1186/s12885-018-4479-2

**Published:** 2018-05-10

**Authors:** Michele Guida, Stefania Tommasi, Sabino Strippoli, Maria Iole Natalicchio, Simona De Summa, Rosamaria Pinto, Antonio Cramarossa, Anna Albano, Salvatore Pisconti, Michele Aieta, Ruggiero Ridolfi, Amalia Azzariti, Gabriella Guida, Vito Lorusso, Giusepe Colucci

**Affiliations:** 1 Medical Oncology Department, National Cancer Research Centre “Giovanni Paolo II”, Via O. Flacco, 65, 70124 Bari, Italy; 2 Molecular Genetics Laboratory and Radiology, National Cancer Research Centre “Giovanni Paolo II”, Via O. Flacco, 65, 70124 Bari, Italy; 3 Laboratory of Molecular Oncology of Solid Tumors and Pharmacogenomics, Ospedali Riuniti, Viale Pinto, 1, 71122 Foggia, Italy; 4 Radiology Department, National Cancer Research Centre “Giovanni Paolo II”, Bari, Italy; 5 Medical Oncology Department, San Giuseppe Moscati Hospital, Via per Martina Franca, 74010 Statte, Taranto Italy; 6 Medical Oncology Department, National Institute of Cancer, Via Padre Pio, 1. 85028 Rionero in Vulture, Potenza, Italy; 7 Medical Oncology Department, National Cancer Institute of Romagna (IRST), Via Piero Maroncelli, 40. 47014 Meldola, Forlì, Italy; 8 Clinical and Preclinical Pharmacology Laboratory, National Cancer Research Centre “Giovanni Paolo II”, Via O. Flacco, 65, 70124 Bari, Italy; 9 Department of Basic Medical Sciences, Neurosciences and Sense Organs, University of Bari, Piazza Giulio Cesare, 1, 70124 Bari, Italy

**Keywords:** Melanoma, Chemotherapy, Base excision repair, MGMT, Fotemustine, Temozolomide, Biomarkers

## Abstract

**Background:**

It is frequently asked whether chemotherapy can still play a role in metastatic melanoma considering the effectiveness of the available drugs today, including antiCTLA4/antiPD1 immunotherapy and antiBRAF/antiMEK inhibitors. However, only approximately half of patients respond to these drugs, and the majority progress after 6–11 months. Therefore, a need for other therapeutic options is still very much apparent.

We report the first large trial of a sequential full dose of fotemustine (FM) preceded by a low dose of temozolomide (TMZ) as a chemo-modulator in order to inactivate the DNA repair action of O(6)-methylguanine DNA-methyltransferase (MGMT). Primary endpoints were overall response and safety. We also evaluated specific biological parameters aiming to tailor these chemotherapies to selected patients.

**Methods:**

A total of 69 consecutive patients were enrolled. The main features included a median age of 60 years (21–81) and M1c stage, observed in 74% of the patients, with brain metastases in 15% and high LDH levels in 42% of the patients. The following schedule was used: oral TMZ 100 mg/m^2^ on days 1 and 2 and FM *iv* 100 mg/m^2^ on day 2, 4 h after TMZ; A translational study aiming to analyse MGMT methylation status and base-excision repair (BER) gene expression was performed in a subset of 14 patients.

**Results:**

We reported an overall response rate of 30.3% with 3 complete responses and a disease control rate of 50.5%. The related toxicity rate was low and mainly of haematological types. Although our population had a very poor prognosis, we observed a PFS of 6 months and an OS of 10 months. A non-significant correlation with response was found with the mean expression level of the three genes involved in the BER pathway (APE1, XRCC1 and PARP1), whereas no association was found with MGMT methylation status.

**Conclusion:**

This schedule could represent a good alternative for patients who are not eligible for immune or targeted therapy or whose previous therapies have failed.

**Trial registration:**

EUDRACT 2009–016487-36l; date of registration 23 June 2010.

**Electronic supplementary material:**

The online version of this article (10.1186/s12885-018-4479-2) contains supplementary material, which is available to authorized users.

## Background

Malignant melanoma, although far less prevalent than non-melanoma skin cancers, is the major cause of death from cutaneous neoplasms. MM remains a cancer with a poor prognosis and a chemoresistance profile. However, since 2011, an improvement in overall survival has been obtained thanks to major advances in understanding the driver molecular alterations and the immunogenic potentiality of this unique cancer [1]*.* The selective inhibitors vemurafenib and dabrafenib, alone or in combination with MEK inhibitors, have achieved a response rate of approximately 50–70%, resulting in improved progression-free survival (PFS) and overall survival (OS) as shown in Phase III studies of patients harbouring BRAF mutations [2, 3]*.* Nevertheless, a high rate of G3-G4 toxic events ranging from 48 to 63% has also been reported with approximately 15% of patients discontinuing treatment due to side effects. In addition, the majority of patients progressed after approximately 12 months because of the occurrence of numerous mechanisms of resistance to anti-BRAF/MEK drugs [2, 3].

In the immune-therapy field, the immunomodulating antibodies that target the checkpoints CTLA-4 (ipilimumab) and PD1 (nivolumab and pembrolizumab) alone or in combination showed survival benefits as both first and second line therapies. The response rate and the PFS ranged from 15% and 2 months, respectively, for ipilimumab [4] to approximately 40% and 6 months, respectively, for antiPD1. The combination of these drugs resulted in a significant increase in the response rate to 60% with a PFS of approximately 12 months, but its toxicity profile was often unacceptable with G3-G4 side effects reported for over 50% of patients and with therapy interruption in approximately 40% of them [5, 6].

Parallel to the spread of its use, for immunotherapy, many escape mechanisms have been reported so that only a few patients are long-term survivors [7, 8].

Therefore, a considerable number of MM patients receive standard chemotherapy mainly as a subsequent line of therapy. The need to define novel therapeutic strategies that overcome the chemotherapy resistance of MM is still relevant today and represents one of the main challenges in the treatment of advanced disease.

Active chemotherapies in MM include alkylating agents such as dacarbazine (DTIC), temozolomide (TMZ) and fotemustine (FM). DTIC gives an overall response rate of only 10–15% with a complete response in less than 5% of patients and a survival of 7–8 months [9]. Similar overall response rates were achieved with both TMZ and FM. The first drug has a high oral bioavailability with an extensive tissue distribution [10], and the latter has good penetration through the blood-brain barrier but relevant myelotoxic side effects [11].

The activity of alkylating agents depends on their capacity to form alkyl adducts that are made by a chloroethyl group being added to the DNA nucleotide guanine in the case of FM. This action results in DNA interstrand cross-links, which in turn trigger the apoptotic cascade. However, the antineoplastic activity of these agents is limited by cellular resistance principally induced by the DNA repair enzyme O(6)-methylguanine DNA-methyltransferase (MGMT), which removes the chloroethyl group from the DNA strands before the crosslink is established [12].

The depletion of MGMT can reverse resistance to alkylating agents and seems to be induced by continuous drug administration as documented in laboratory research and clinical trials [12–15]*.*

To date, the use of TMZ as a chemo-modulating agent has never been tested in an MM patient population. We evaluated this hypothesis in a feasibility study that included two cohorts of patients treated with two schedules of TMZ (100 mg/m^2^ over 2 days) in combination with FM (100 mg/m^2^ on the second day 4 h after TMZ) in order to identify the optimal doses and timing of administration according to an acceptable safety profile and a strong antitumour activity [16]. We found that this chemotherapy regimen was better tolerated in terms of myelotoxicity when it was administered on a schedule of day 1–21 rather than on days 1 and 8 every 21 days [16, 17].

Thus, we planned a new multicentre phase II trial to verify the effectiveness of this treatment schedule in a larger population of patients. Moreover, we attempted to build a translational study by evaluating a posteriori some biological parameters implicated in drug resistance in order to unearth candidate novel biomarkers that are suitable as predictive and prognostic tools to help us identify responsive patients and optimize the use of these “old” drugs.

## Methods

### Patient population

We enrolled 69 patients with metastatic melanoma not previously treated with chemotherapy. Eligible patients were 18 years old or older with measurable lesions (according to the RECIST criteria), an Eastern Cooperative Oncology Group (ECOG) performance status of ≤2, a life expectancy of more than 12 weeks as well as adequate renal, hepatic and bone marrow functions. Patients with asymptomatic or symptomatic brain metastases were admitted on the condition that they had brain disease stabilized by previous loco-regional treatments and no additional disease sites. The study was conducted in accordance with the international standards of good clinical practice. The protocol was approved by the local Ethics Committee of National Cancer Research Centre “Giovanni Paolo II”, Bari, Italy. The date of registration was June 2010, and the first patient was enrolled in June 2010. The period of accrual was from June 2010 to October 2013.

The main patient features are listed in Table 1. Genetic evaluation of the BRAF mutation status was performed in 41 patients (59% of patients). Our population was unbalanced towards wild-type BRAF because targeted therapy is available. Therefore, genetic evaluation became paramount, and in the present study, we enrolled almost exclusively patients with wild-type BRAF. According to the AJCC melanoma staging system, 74% (51) had M1c with 15% with brain metastases.

**Table 1:** Baseline characteristic of patients

Age-yr	
Median	60
Range	21–81
Sex-no. (%)	
Male	39 (56,5)
Female	30 (43,5)
ECOG performance status- no.(%)	
0	20 (29)
1	37 (53)
2	12 (18)
Site of primitive melanoma- no.(%)	
skin	58 (84)
uveal	3 (4)
mucosal	2 (3)
unknown	6 (9)
Melanoma stage-no.(%)	
M1a	5 (8)
M1b	13 (18)
M1c	51 (74)
Site of metastases-no.(%)	
1	16 (25)
2	19 (27)
≥ 3	34 (48)
Brain metastases-no.(%)	
yes	10 (15)
no	59 (85)
BRAF status-no. (%)	
Wild type	31 (46)
BRAF V600	7 (13)
BRAF not V600	3 (2)
Unknown	28 (41)
Prior adjuvant therapy no.(%)	
vaccine	2 (3)
interferon	13 (19)
none	54 (47)
Disease free survival-months	
Median	13
Range	0–136
Basal level of LDH (normal range 240–480 mg/dl)	
High	29 (42)
Normal	40 (58)

#### Treatment

The treatment schedule provided TMZ orally administered at a dose of 100 mg/m^2^ on days 1 and 2 and by intravenous FM at a dose of 100 mg/m^2^ on day 2, 4 hours after TMZ. The treatment cycle was repeated every 21 days until progression or up to 9 cycles.

The National Cancer Institute Common Terminology Criteria for Adverse Events, version 4.0 (NCI CTCA) was used to grade toxicity.

#### Clinical evaluation

The prestudy evaluation was completed within 2 weeks before receiving the study drugs. Response Evaluation Criteria In Solid Tumors (RECIST version 1.1) criteria was used for efficacy assessment. Tumour assessments were obtained at screening and at the end of every three cycles (approximately every 12 weeks).

### Biological study

#### MGMT promoter methylation

DNA was extracted from FFPE cancer tissue (n. 14 patients) containing at least 70% tumour cells and from normal skin tissues using the QIAamp DNA FFPE Tissue Kit (Qiagen) according to the manufacturer’s protocol. The percentage methylation was automatically calculated by the PyroMarl CpG software (Biotage/Qiagen). Ten CpG sites in the MGMT gene promoter region (chr10:131,265,507–131,265,556) were assessed.

#### RNA extraction, cDNA synthesis and quantitative real-time PCR

RNA was extracted from 14 malignant and 3 non-tumoural FFPE samples (healthy dermis) with the RNeasy® Plus Mini Kit (Qiagen) as indicated by the manufacturer and quantified with NanoDrop8000 (Thermo Scientific). Probes were directed against REF1/APEX1 (Hs00172396_m1), XRCC1 (Hs00959834_m1) and PARP1 (Hs00242302_m1) (see Additional file 1: Methods).

##### End point and statistical analysis

The primary endpoint was the tumour response evaluation. The trial was designed to assess whether the activity of the treatment schedule determined an ORR (complete response [CR] plus partial response [PR]) of not less than 12% (cut-off for considering the treatment was not active) and assumed that 25% was the minimum expected response for a combination with good activity. With the aim of blocking the study in an early stage (interim analysis), if the ORR was lower than the value indicated by the cut-off, a Simon’s two-stage design was used. The null hypothesis that the true response rate was 0.12 was tested against a one-sided alternative. In the first stage, 19 patients were accrued. If there were 2 or fewer responses in these 19 patients, the study was stopped. Otherwise, 42 additional patients were accrued for a total of 61. The null hypothesis was rejected if 12 or more responses were observed in 61 patients. This design yielded a type I error rate of 0.2 and a power of 0.8 when the true response rate was 0.25.

The secondary objectives included the evaluation of PFS, OS, and the response duration and the assessment of the safety profile as well as of the response by predictive biomarkers.

For the latter purpose, a statistical analysis of the original continuous expression data was performed using a Mann-Whitney test. Patients were stratified according to gene expression status (high and low expression) considering median relative expression as the cut-off, and we compared them taking into account PFS with the Kaplan-Meyer method. The final statistical analysis was conducted in November 2015.

## Results

### Clinical results

**Fig. 1:**
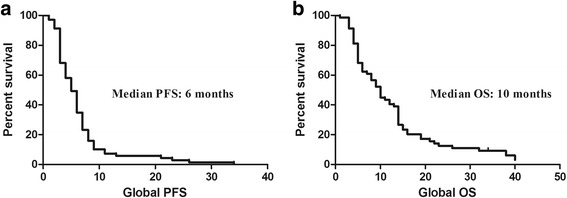
Kaplan Meyer curves for global PFS (**a**) and OS (**b**)

**Fig. 2:**
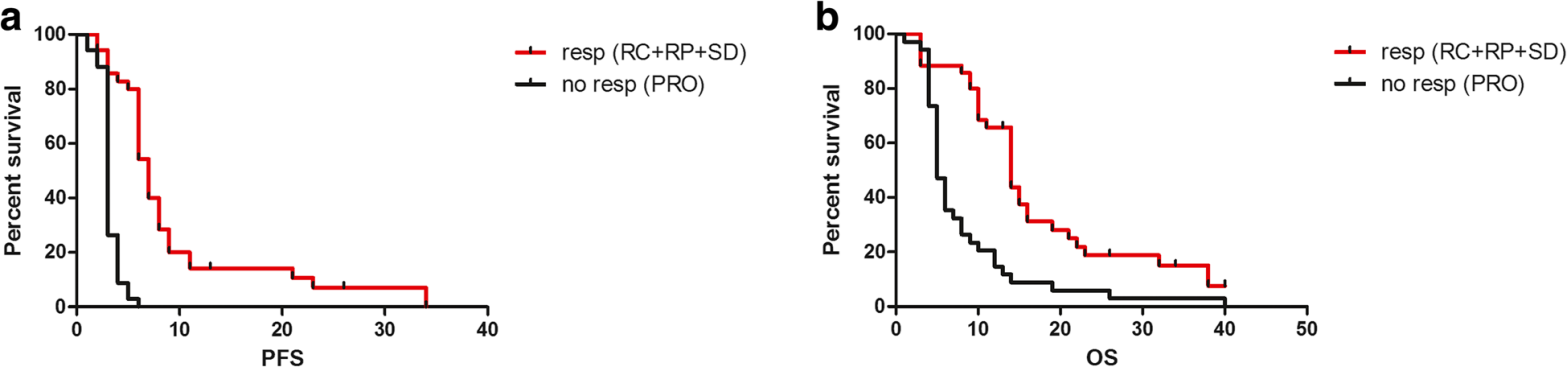
Kaplan Meyer curves for global PFS (**a**) and OS (**b**) for responsive patients (no. 35 red line) vs non responsive patients (no. 34 black line). CR: complete response; PR: partial response; SD: stable disease; PRO: progressive disease

All enrolled patients received a median of 5 cycles of treatment (range 1–9). Globally, an ORR was obtained for 21 patients (30.3%), including 3 CRs and 18 PRs with a median response duration of 5 months (2–31). In addition, a further 14 patients obtained SDs with an overall clinical benefit (CR + PR + SD) of 50.5%. Regarding the secondary end points, the median PFS was 6 months (2–34), and the median OS was 10 months (2–40+) (fig. 1). When we compared PFS and OS in responsive/SD patients (35 patients) vs non-responsive patients (34 patients), we noted significant differences in terms of the median PFS (7 vs 3 months) and median OS (14 vs 5 months) (fig. 2).

Notably, in the small group of BRAF V600 patients, there were no differences in ORR compared with the largest group of wild-type patients. It is noteworthy that a CR was achieved in the patient with BRAF K601E, while the patients with BRAF G469A and D594G reached a PR with response durations of 26, 11 and 4 months.

Thirty-two of the 69 patients, after this first-line chemotherapy trial, received subsequent treatments including ipilimumab (26%), nivolumab (1 patient), vemurafenib (10%) and other chemotherapy (10%). Only 10 of them obtained any brief clinical control from these further treatments, so they did not influence the median survival of our entire population.

#### Safety and dose delivery

The toxicity profile was evaluated on 323 cycles of therapy delivered. The median of delivered cycles was 5 (1–9).

The present study confirmed an acceptable toxicity profile as already reported in our previous feasibility study. The main side effects are reported in Table 2.

**Table 2:** Treatment-related adverse events that occurred in at least one of the enrolled patients

Event	All grade-no.(%)	Grade 3–4-no.(%)
Neutrophil count decreased	21(30)	5 (7)
Platelet count decreased	23 (33)	5 (7)
Anemia	12 (17)	2 (2)
Alopecia	2 (2)	/
Diarrhea	1 (1)	/
Nausea	8 (12)	2 (2)
Vomiting	5 (7)	2 (2)

The most frequent adverse events (AEs) were haematological mainly in terms of thrombocytopenia and neutropenia, which occurred as G3 and G4 in only 7% of patients.

### Biological assessment

The MGMT gene promoter was methylated in all 14 patients with a range of methylation of 6–13%. No association was present between the methylation level of the promoter region of MGMT for any of the 10 CpG sites or the clinical outcomes of the patients. In contrast, the analysis of genes involved in base-excision repair (BER) showed that the mean expression level of the three genes (APE1, XRCC1 and PARP1) was higher in patients who did not respond to therapy (Table 3).

**Table 3:** Expression analysis of genes involved in base-excision repair

	APE1 Median (range)	PARP1 Median (range)	XRCC1 Median (range)
Stable disease/Partial response (*n* = 7)	32.45 (0.01–176.7)	0.5 (0.001–2.63)	0.2 (0.008–1.05)
Progression (n = 7)	47.18 (4.48–87.73)	1.36 (0.01–26.17)	0.58 (0.04–1.26)

Data are expressed as relative log2 expression

Moreover, we stratified patients according to gene expression status (up- and downregulation considering median relative expression as a cut-off) and analysed them with respect to PFS.

**Fig. 3:**
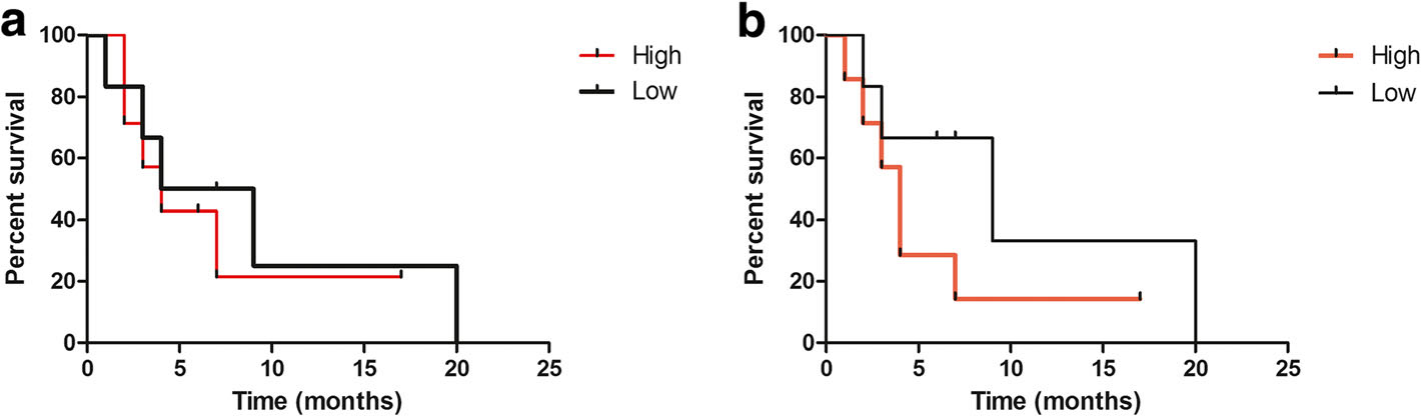
Kaplan Meyer curves for PFS in patients relative to PARP1 (**a**), and XRCC1 (**b**) expression levels

Kaplan-Meyer curves showed a longer median PFS for patients with downregulation of PARP1 (6.5 versus 4 months), XRCC1 (9 versus 4 months) and APE1 (9 versus 7 months) (Fig. 3). Statistical analyses did not show any significant biological assessment results due to the small sample size.

## Discussion

It has been frequently asked whether there is a role for chemotherapy in MM considering the numerous drugs available today. The response rates to combination target or immune-therapy with antiBRAF/antiMEK and antiCTLA4/antiPD1 range from 58 to 69%, and the disease control rate is 75% of patients receiving both of these therapies. However, most patients progress after approximately 12 months of treatment, and only a few of them achieve long-term control of their disease. Moreover, the toxicity profile of these new drugs is often unacceptable with G3-G4 side effects reported for over 50% of patients, causing many to discontinue the drugs. Finally, these drugs are often not indicated for patients with various types of comorbidities such as autoimmune, ocular and cardiac diseases. Therefore, the need for other therapeutic options is still very important.

We conducted the first large clinical study for MM, aiming to explore the effectiveness of sequential non-therapeutic, chemo-modulating low doses of TMZ after a full dose of FM. Currently, few data are available, and no dosing or schedules have been established. Additionally, the optimal interval between the administration of the two drugs is not yet clear. A depletion in MGMT can be gained in melanoma cells when TMZ is administered at a low dose of 100–200 mg/m^2^ consecutively for 2 days. This enzymatic deficiency can amplify the effectiveness of FM when it is given on the second day approximately 4 h after TMZ [12–14]*.* In MM, two previous studies have tested the combination of TMZ with nitrosureas, namely, FM [17] and lomustine [18]. In both of these trials, TMZ was given at a higher dose than our schedule and with an additive/synergistic intent in combination with the full dose of nitrosureas. As a consequence, an unacceptable toxicity with a higher rate of myelotoxicity was reported in both studies. In particular, Tas et al. [17] reported a dose reduction in 45% of the patients, a dose delay in 32.5%, with a toxicity related discontinuation of 27.5%, a response rate of 35% and a low median survival of only 6.7 months.

We used a regimen previously verified in our pilot study [16]*.* In the present study, in a large cohort of 69 MM patients, we confirmed a response rate of 30.3% and an overall clinical benefit of 50.5%. The median PFS was 6 months, and the median OS was 10 months. Notably, our patient population included 74% patients in the M1c stage, of whom 15% had brain metastases. This means that this population had a very poor prognosis.

When we compared patients who obtained a clinical benefit (SD + PR + CR) versus patients with progressive disease, we found a median PFS of 7 versus 3 months and a median OS of 14 versus 6 months. These data mean that a huge effort should be made to tailor these drugs to selected patients through the identification of biomarkers.

In our previous proteomic study carried out in 20 patients of this same population, we identified some peptides that were significantly upregulated in responder patients and associated with proteins involved in the control of redox cellular homeostasis, such as NQO1, and in the regulation of apoptosis, such as RIN1 [19].

In this translational effort, we also explored if the effectiveness of our schedule was predicted by the level of MGMT methylation. We found low levels (6–13%) of pretreatment MGMT methylation, which were not related to the clinical response. Our findings are in accordance with previous data showing that in MM no association exists between the clinical response to chemotherapy and basal levels of MGMT [20–22]. Otherwise, it has been reported that low MGMT nuclear expression, evaluated by immunohistochemistry, is associated with better outcomes only in patients with BRAF mutations treated with a cisplatin, vinblastine and temozolomide regimen as the first-line therapy [23], and in glioblastoma, MGMT methylation greater than 35% has been described as an independent prognostic factor associated with better outcomes [24, 25].

Notably, MGMT is not an exclusive player involved in melanoma cell death induced by alkylating drugs [12, 25]. The inherent deficiency of the downstream apoptotic pathway might be a key resistance mechanism, and this might be due to several sources such as mismatch repair protein inactivation and alterations in DNA damage repair pathways.

Thus, we analysed the expression of genes involved in the base-excision repair (BER) pathway because of their emerging importance in enhancing the cytotoxicity of DNA damaging agents (e.g., alkylating agents). We were able to collect FFPE samples for only 14 patients before treatment. Notwithstanding the small sample size, gene expression of APE1, XRCC1 and PARP1 was measured to verify a trend that could explain the response to treatment. APE1 is involved in a key step of BER and has an almost unique role in the processing of apurinic/apyrimidinic sites [26]. We observed that the basal mean level of APE1 gene expression was elevated in patients who did not respond to treatment versus those who responded to chemotherapy. Abbots et al. [27] reported that APE1 inhibition is efficient in PTEN-deficient melanoma cell lines, and our results encouraged us to further investigate the role of this enzyme in MM. In a similar way, we observed the upregulation of protein 1 of the PARP family in patients who progressed after a few cycles of TMZ/FE treatment. Moreover, patients with PARP1 downregulation showed a longer median OS rate.

XRCC1 is a scaffold protein with no enzymatic activity that interacts with several components of the BER pathway. Its deficiency is responsible for mutations and a high rate of sister chromatid exchange, which leads to genomic instability. It has been reported that such a deficiency results in chemo-sensitivity [28]. Abdel-Fatah et al. [29] reported that a deficiency in XRCC1 in ovarian cancer is associated with a clinical response to cisplatin treatment. In accordance with this study, we observed a slightly elevated mean expression level in non-responding patients, although the survival analysis showed that patients with upregulated expression had a longer median OS rate. This result seems to confirm that of another study reporting that wild-type XRCC1 cell lines are more sensitive to TMZ and, more interestingly, that effective PARP inhibition requires a functional XRCC1 protein [30].

Although increased expression of BER genes we observed in the not-responding patient group was not significant, our preliminary results encourage verification of the role of players in the BER pathway in melanoma treatment both as predictive biomarkers, such as XRCC1, and as molecular targets (PARP1 or APE1) in order to enhance current therapeutic settings.

## Conclusion

In this large phase II trial, we demonstrated that the combination of two “old” alkylating agents effectively works in terms of both overall response and survival with an acceptable toxicity profile. In view of the increasing range of therapeutic options now available, an emerging challenge for clinicians is to establish a useful algorithm of sequential treatment for MM patients. Chemotherapy can still play a role, mainly in BRAF wild-type patients who progress on immune therapy or for whom immunotherapy is contraindicated. Additionally, in patients with mutated BRAF, chemotherapy can be utilized mostly in cases of fast progression during targeted therapy. For this purpose, we must endeavour to shed light on the mechanisms underlying drug responses and resistance as well as to outline useful biomarkers that could help us to tailor the optimal agents to the appropriate subjects at the right time.

## Additional file

Additional file 1: The methods of quantitative Real-Time PCR evaluation of the genes of BER and MGMT promoter methylation assessment are described in the additional file. (DOCX 13 kb)

## Abbreviations

AEs: Adverse events
AJCC: American Joint Committee on Cancer
APE1: Apurinic/apyrimidinic endonuclease
BER: Base-excision repair
BRAF: v-Raf murine sarcoma viral oncogene homolog B
CR: Complete response
CTLA4: Cytotoxic t-lymphocyte antigen 4
DTIC: Dacarbazine
ECOG: Eastern Cooperative Oncology Group
FFPE: Formalin-fixed, paraffin-embedded
FM: Fotemustine
MEK: Mitogen-activated protein kinase
MGMT: O(6)-methylguanine DNA-methyltransferase
MM: Metastatic melanoma
NCI CTCAE: National Cancer Institute Common Terminology Criteria for Adverse Events
NQO1: NAD(P)H dehydrogenase, quinone 1
ORR: Overall response rate
OS: Overall survival
PARP1: Poly ADP-ribose polymerase 1
PD: Progressive disease
PD1: Programmed death 1 receptor
PFS: Progression free survival
PR: Partial response
RECIST: Response Evaluation Criteria in Solid Tumors
RIN1: Ras and Rab interactor 1
SD: Stable disease
TMZ: Temozolomide
XRCC1: X-ray repair cross-complementing protein 1
