# Supplementary material for: The search for a melanoma-tailored chemotherapy in the new era of personalized therapy: a phase II study of chemo-modulating temozolomide followed by fotemustine and a cooperative study of GOIM (Gruppo Oncologico Italia Meridionale)
Source: BMC Cancer. 2018 May 10;18:552. doi: 10.1186/s12885-018-4479-2 (PMC5946485; doi:10.1186/s12885-018-4479-2)
Supplement: Supplementary file 1 — The methods of quantitative Real-Time PCR evaluation of the genes of BER and MGMT promoter methylation assessment are described in the additional file. (DOCX 13 kb) [file 12885_2018_4479_MOESM1_ESM.docx]

*quantitative Real-Time PCR evaluation of genes of BER*

RNA extracted from healthy tissues were mixed in equimolar fashion in order to obtain pools of RNA which were useful to normalize data. 500 ng of RNA were retro-transcribed through the High-Capacity cDNA Reverse Transcription Kit (Applied Biosystems) as indicated by the manufacturer.

Analysis of expression was performed using a fluorescence-based, real-time detection method (TaqMan probes; Applied Biosystems) on the ABI PRISM 7000 Sequence Detection System (Applied Biosystems). The selected assays were designed across an exon-exon junction to produce a short amplicon which gives a more efficient PCR reaction. A 20 µl PCR reaction including 2.5 µl of RT product, 1X TaqMan Universal PCR Master Mix and 1X of the corresponding TaqMan Gene Expression Assay was incubated in 96-well plates at 95°C for 10 min followed by 40 cycles of 95°C for 15 s and 60°C for 1 min. PCR reactions were performed in triplicate including no-template controls. Relative quantities of each cDNA were calculated using the ΔΔCt method after normalization with endogenous reference 18s rRNA (Hs03928985_g1) and calibrating Ct values with respect to the Ct of healthy tissues.

MGMT promoter methylation assessment

Promoter methylation status of the *MGMT* gene was conducted with a polymerase chain reaction (PCR)-based method using DNA treated with bisulfite followed by a real-time pyrosequencing that targeted CpG islands. This method allowed us to quantify methylation at multiple CpG sites individually. Pyrosequencing was performed using PyroGold Q96 SQA Reagents and the Pyro Q-CpG software on a PyroMark ID pyrosequencer (Biotage AB, Sweden) as per the manufacturer’s recommendations. The sequencing results were analysed using the PSQ PyroMark software (Biotage AB). As controls, Methylated Control Dna (positive methylation control), and Non- Methylated Control Dna (negative methylation control) were included in the assay, as well as a reaction without any template DNA (non-template control). All tumour and control specimens were measured in triplicates.
